# HIV seronegativity in children, adolescents and young adults living with perinatally acquired HIV: A cross‐sectional study in Thailand

**DOI:** 10.1002/jia2.25614

**Published:** 2020-09-23

**Authors:** Praew Wirotpaisankul, Keswadee Lapphra, Alan Maleesatharn, Supattra Rungmaitree, Orasri Wittawatmongkol, Wanatpreeya Phongsamart, Nantaka Kongstan, Benjawan Khumcha, Kulkanya Chokephaibulkit

**Affiliations:** ^1^ Department of Paediatrics Faculty of Medicine Siriraj Hospital Mahidol University Bangkok Thailand

**Keywords:** adolescents, children, HIV, seronegative, perinatally‐acquired HIV, Thailand

## Abstract

**Introduction:**

Early initiation of combination antiretroviral therapy (ART) with long‐term viral suppression may lead to seronegativity in grown‐up children with perinatally acquired HIV (PHIV). This study aimed to determine the frequency and associated factors of seronegativity in Thai children, adolescents and young adults with PHIV.

**Methods:**

A cross‐sectional HIV serological study was performed in children, adolescents and young adults two years or older who were receiving ART with undetectable HIV‐RNA for at least one year from August 2018 to August 2019. Medical records were extracted for multivariate analysis of independent factors for seronegativity.

**Results and discussion:**

Of 110 patients, 50 male, median (range) age was 18.4 (4.8 to 26.6) years, 8 (7.3%) were seronegative, and 1 (0.9 %) was inconclusive. The seronegative group had a younger median (range) age at ART initiation: 3.0 (1.0 to 12.0) versus 40.0 (2.0 to 207.0) months, *p* = 0.045; and shorter median (range) duration from ART initiation to viral suppression: 16.8 (7.2 to 42.0) versus 55.2 (6.0 to 214.8) months, *p* = 0.036. Multivariate analysis identified younger age at ART initiation (aOR 0.69, 95% CI 0.49 to 0.98, *p* = 0.038) and shorter time to viral suppression after ART initiation (aOR 0.94, 95% CI 0.89 to 0.99, *p* = 0.019) as independent factors associated with HIV seronegativity. Of the infants who initiated ART < 3 and between three and six months of age, 50% and 26.7% became seronegative respectively.

**Conclusions:**

HIV seronegativity was observed in children and adolescents with PHIV who initiated ART early in infancy and had rapid and sustained virological response. Awareness of this phenomenon will help avoid inappropriate treatment interruption on the basis of negative antibody testing.

## 1 INTRODUCTION

There are increasing reports of infants who initiated very early combination antiretroviral therapy (ART) remaining serology negative for human immune deficiency virus (HIV) or subsequently undergoing HIV seroreversion [1, 2, 3, 4]. HIV seroreversion is defined as a decrease in the level of antigen and/or antibody below the cut‐off value of the test, turning a once positive result negative [5]. HIV seroreversion can occur in HIV negative infants following the waning of maternal antibodies transmitted in utero, neonates with HIV who initiate very early effective ART, individuals initiating ART during acute infection, and among people with late‐stage AIDS [1, 6, 7, 8].

Since 2010, the World Health Organization (WHO) has recommended the initiation of ART for all children under two years of age diagnosed with HIV infection, regardless of the severity of disease or CD4^+^ T lymphocytes (CD4) counts [9]. Several reports have shown that there is an association between early initiation of ART and HIV seronegativity in infants [1, 10, 11]. Given the widespread availability of infant HIV diagnosis at birth and antiretroviral treatment, this may lead to an increasing number of children living with HIV who are seronegative. This is clinically relevant as a negative HIV serology result could lead to an incorrect diagnosis and inappropriate cessation of ART.

The new fourth‐generation HIV serological tests include the detection of p24 antigen allowing very early detection of HIV acquisition. Also, these newer assays are more sensitive which could result in a longer time to detect seroreversion in HIV‐exposed uninfected infants [12]. Today, there are limited data on the use of fourth generation assays among children receiving suppressive ART.

The aim of this study was to determine the frequency of seronegativity or seroreversion in children, adolescents and young adults with perinatally acquired HIV (PHIV) who have been receiving effective ART with undetectable HIV‐1 RNA (viral load; VL) and the factors that potentially contribute to seronegativity or seroreversion.

## 2 METHODS

### 2.1 Study design and participants

A single‐centre cross‐sectional study was conducted at the paediatric HIV clinic, Department of Paediatrics, Siriraj Hospital, Mahidol University, Bangkok, Thailand, from August 2018 to August 2019. Children, adolescents and young adults with HIV since childhood, confirmed by two positive HIV DNA PCR assay in infancy, and/or high HIV RNA (>10,000 copies/mL), and/or with AIDS defining condition(s); who were receiving ART and had viral suppression (VL < 50 copies/mL) for at least one year (based on at least two consecutive measurements), were invited to participate in the study. Only children perinatally infected with HIV were included. Perinatally acquired infection was based on history of maternal HIV‐infection and no other evidence of alternative modes of HIV acquisition. The primary objective was to determine the rate of negative HIV serology and the secondary objective was to identify factors associated with HIV seronegativity.

### 2.2 Procedures

The study was reviewed and approved by the Siriraj Institutional Review Board, Faculty of Medicine Siriraj Hospital, Mahidol University, Certificate of approval SIRB Protocol No: 250/2561(EC2). After informed consent, and assent in children aged between seven and eighteen years, a single blood drawn was taken to test for HIV serology.

Case record forms were filled using data extracted from medical records. Per standard of care, VL was checked every six months during the first year of treatment and then every 12 months thereafter if virologically suppressed. CD4 cell counts were monitored every six months. Data collected included: demographic data, medical history including prenatal and perinatal history, maternal ART use, age of ART initiation, CD4 cell counts at the time of diagnosis, CD4 cell counts on the day of enrolment (or within one year if result missing), nadir CD4 cell counts, duration of ART, time from ART initiation to first report of viral suppression, duration of viral suppression, history of viral blip, ART regimens received, and ART compliance.

The HIV serological test was performed at the hospital’s central lab using fourth‐generation test kits (Abbott^®^ Architect i2000, IL, USA; Roche^®^ Cobas E601, IN, USA; Vidas^®^ PC, Marcy l’Étoile, France). If the HIV serology test result was inconclusive, the test was repeated six months after the first test.

### 2.3 Definitions

HIV serology results were reported as positive, negative and inconclusive. Repeated inconclusive test results were included in the HIV seronegative group for the data analysis. Gestational age was divided into term and preterm. Infants were defined term if their gestational age was ≥37 weeks at delivery and preterm if their GA was <37 weeks. Good ART adherence referred to those who self‐reported no missed doses in the last three clinic visits. A viral blip was defined as intermittent viraemia with VL between 50 and 999 copies/mL after the subject had already been virologically suppressed. ART regimens were divided into three groups; NNRTI (non‐nucleoside reverse transcriptase inhibitors), PI (protease inhibitors) and INSTI (integrase strand transfer inhibitor) containing regimens.

### 2.4 Analysis

The rate of HIV seropositivity and seronegativity were calculated. Associated factors were compared between the seropositive and seronegative groups using logistic regression. Variables with *p* < 0.1 in the univariate model were included in the stepwise multivariate model. All analyses were carried out using Stata® software version 11.2 (StataCorp 4905 Lakeway Drive, College Station, TX, USA).

## 3 RESULTS AND DISCUSSION

A total of 115 children were enrolled. Five children were excluded, one child due to a detectable viral load at enrolment and four children after a review of medical records found that their mode of HIV acquisition was sexual. Among 110 patients, 101 (91.8%) were HIV seropositive, eight (7.3%) were seronegative, and one (0.9%) was inconclusive (repeated). Details of the nine (8.2%) seronegative child are shown in Table 1.

**Table 1:** Characteristics of nine children and adolescents living with perinatally acquired HIV who are HIV seronegative or inconclusive

No. Sex	HIV serology	Worst clinical Staging	Timing of HIV PCR (months of age)	Birth weight (g) and mode of delivery	Maternal gestational age at ART initiation	Maternal VL (copies/mL)	Maternal CD4 (cells/µL)	CD4 at cART initiation (cells/µL, %)	HIV viral load at ART initiation (copies/mL)	Age at start ART (months)	Duration of ART (years)	Time from cART initiation to VL suppression (months)	Nadir CD4 (cells/µL)	Duration of viral load suppression (years)
1.M	Neg	C1	birth, 1, 2	3300 C/S	AZT (34 weeks)	5000	N/A	1366 (33.7%)	56,400	1	10.2	36	876	7.3
2.M	Neg	C3	2, 6	2800 C/S	AZT (32 weeks)	N/A	300	778 (12.6%)	2,770,000	3	9.1	42	642	5.6
3.F	Neg	C3	3	2440 NL	N/A	N/A	N/A	220 (11.2%)	>10,000,000	3	8.7	11	220	7.8
4.F	Neg	N1	1, 2	2630 NL	N/A	N/A	N/A	2016 (35%)	1,592,288	4	15.1	19	619	13.5
5.F	Neg	C3	4, 6	2510 NL	N/A	N/A	N/A	732 (15.3%)	750,000	12	15.5	11	635	14.6
6.M	Neg	B1	1, 4	3240 C/S	TDF/3TC/LPV/r (34 weeks)	289	462	2709 (41.7%)	1,820,000	2	4.7	17	479	3.3
7.F	Neg	C3	birth, 1, 2	3130 NL	N/A	N/A	N/A	259 (18%)	N/A	10	16.3	10	259	15.5
8.M	Neg	N1	birth, 4	2680 NL	N/A	N/A	N/A	2029 (40.3%)	N/A	1	12.8	7	606	12.2
9.F	Inconclusive	B1	4 (and positive HIV serology at two years of age)	1950 NL	N/A	N/A	N/A	3113 (27.4%)	N/A	5	12.8	24	855	10.8

3TC, Lamivudine; ART, combination antiretroviral therapy; AZT, Zidovudine; C/S, Caesarean section; LPV/r, Lopinavir/Ritonavir; N/A, missing data; NL, normal labour; NVP, Nevirapine; TDF, Tenofovir; VL, viral load;

At enrolment, the median (range) age in the seronegative children was lower compared to the seropositive children, 12.8 (4.8 to 17.2) versus 18.6 (6.7 to 26.6) years (*p* = 0.001), Table 2. Seronegative children also initiated ART at a younger age: 3.0 (1.0 to 12.0) versus 40.0 (2.0 to 207.0) months (*p* = 0.045). The duration from ART initiation to viral suppression was significantly shorter in the seronegative children, 16.8 (7.2 to 42.0) months, compared to 55.2 (6.0 to 214.8) months in the seropositive children (*p* = 0.036). There were no differences in nadir CD4 cell counts, CD4 counts at enrolment or at time of diagnosis. No significant differences between groups were also observed for the duration of viral suppression before testing, presence of viral blip, premature birth, route of delivery, birth weight, maternal antiretroviral treatment before or during pregnancy, adherence to ART, duration of ART and ART regimens.

**Table 2:** Characteristics and factors that may be associated with HIV seronegativity in children, adolescents and young adults living with perinatally acquired HIV

Characteristics	Seropositive	Seronegative	Univariate Analysis		Multivariate Analysis	
	(n = 101)	(n = 9)^b^	Crude OR	*p*‐value	Adjusted OR	*p*‐value
Age at enrolment (years), median (range)	18.6 (6.7 to 26.6)	12.8 (4.8 to 17.2)	0.7 (0.6 to 0.9)	0.001^a^		
Male (%)	46 (45.5)	4 (44.4)	0.9 (0.2 to 3.8)	0.949		
Age at ART initiation (months), median (range)	40.0 (2.0 to 207.0)	3.0 (1.0 to 12.0)	0.80 (0.64 to 0.99)	0.045^a^	0.69 (0.49 to 0.98)	0.038^a^
<3 months; n (%)	3 (3.0)	3 (33.3)	1			
3 to 6 months; n (%)	11 (10.9)	4 (44.5)	0.36 (0.05 to 2.60)	0.314		
>6 months; n (%)	87 (86.1)	2 (22.2)	0.02 (0.003 to 0.19)	0.001^a^		
Time from ART initiation to viral suppression (months), median (range)	55.2 (6.0 to 214.8)	16.8 (7.2 to 42.0)	0.97 (0.93 to 1.00)	0.036^a^	0.94 (0.89 to 0.99)	0.019^a^
<12 months; n (%)	24 (23.8)	4 (44.5)	1			
12 to 36 months; n (%)	13 (12.8)	3 (33.3)	1.4 (0.3 to 7.2)	0.698		
>36 months; n (%)	64 (63.4)	2 (22.2)	0.2 (0.03 to 1.09)	0.062		
Current CD4 T lymphocytes (cells/µL), median (range)	678 (68 to 2307)	820 (479 to 1163)	1.00 (0.99 to 1.00)	0.429		
CD4 T lymphocytes at time of diagnosis (cells/µL), median (range), n = 100 (92/8)^c^	542 (2 to 4037)	1072 (220 to 3113)	1.00 (0.99 to 1.00)	0.089		
Nadir CD4 T lymphocytes (cells/µL), median (range), n = 100 (92/8)^c^	282 (2 to 1707)	619 (220 to 876)	1.00 (0.99 to 1.00)	0.084	1.00 (0.99 to 1.00)	0.275
Duration of viral suppression (years), median (range)	8.3 (0.5 to 16.2)	10.8 (3.3 to 15.5)	1.1 (0.9 to 1.3)	0.230		
Ever have viral blip (%)	40 (39.6)	1 (11.1)	0.2 (0.02 to 1.58)	0.125		
Perinatal history						
Premature delivery (%), n = 103 (94/9)^c^	6 (6.4)	‐	‐	‐		
Normal delivery (vs. Caesarean section) (%), n = 104 (95/9)^c^	74 (77.9)	6 (66.7)	1.7 (0.4 to 7.6)	0.450		
Birthweight (gram), median (range), n = 99 (90/9)^c^	2990 (1310 to 4000)	2680 (1950 to 3300)	0.99 (0.99 to 1.00)	0.417		
Maternal ART started during or before pregnancy (%), n = 90 (82/8)^c^	17 (20.7)	3 (37.5)	2.3 (0.5 to 10.6)	0.287		
Anti‐retroviral treatment and regimens						
No missing ART in the past 3 visits (%)	80 (79.2)	7 (77.8)	0.9 (0.2 to 4.7)	0.919		
Duration of ART (years), median (range)	14.9 (2.8 to 22.2)	12.8 (4.7 to 16.3)	0.9 (0.8 to 1.0)	0.186		
Ever received NNRTI (%)	98 (97.0)	9 (100.0)	‐	‐		
Duration of NNRTI for whom ever received (years), median (range)	10.1 (0.1 to 17.9)	10.7 (1.2 to 16.4)	0.99 (0.87 to 1.13)	0.946		
Ever received PI (%)	46 (45.5)	3 (33.3)	0.60 (0.14 to 2.52)	0.484		
Duration of PI for whom ever received (years), median (range)	10.2 (0.5 to 20.7)	3.5 (1.1 to 8.4)	0.8 (0.5 to 1.1)	0.124		
Ever received INSTI (%)	4 (3.9)	‐	‐	‐		
Duration of INSTI for whom ever received (years), median (range)	3.2 (0.2 to 5.0)	‐	‐	‐		

ART refers to combination antiretroviral therapy that includes at least two classes; NNRTI, non‐nucleoside reverse transcriptase inhibitors; PI, protease inhibitors; INSTI, integrase strand transfer inhibitor.

^a^ Statistically significant *p*‐value.

^b^ HIV seronegative group includes those who had negative and inconclusive HIV serology

^c^ Number in parentheses, numbers of patients in each group with available data.

**Figure 1:**
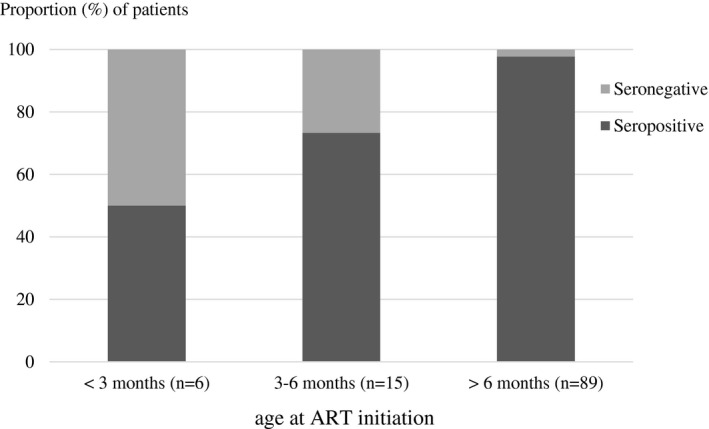
Proportion of HIV serology by age at combination antiretroviral therapy (ART) initiation (%)

In multivariate analysis, a younger age at ART initiation: adjusted odds ratio (aOR) 0.69 (0.49 to 0.98), *p* = 0.038, and a shorter duration from ART initiation to viral suppression: aOR 0.94 (0.89 to 0.99), *p* = 0.019, were independently associated with seronegativity. Among the six children who initiated ART before three months of age, 3/6 (50%) became seronegative. Seronegative rates were lower in children who initiated ART after three months: 4/15 (26.7%) children who initiated ART between three to six months of age were seronegative and 2/89 (2.2%) children who initiated ART after six months were seronegative (Figure 1). All of these subjects remained virologically suppressed since ART initiation.

Several recent reports have shown that seroreversion does occur in children living with HIV who initiate very early ART [1, 2, 3, 4]. After starting ART individuals are not generally retested, therefore data on the prevalence of seroreversion in children and adolescents PHIV is relatively limited. A unique aspect of our study is that we assessed seroreversion in older children/adolescents with PHIV allowing the detection of children who seroreversion later following long‐term viral suppression.

Consistent with previous reports, the seronegative children in our cohort were younger, which is a reflection of the current clinical practice of earlier diagnosis, earlier ART initiation, and improved immunological and virological profiles following treatment initiation.

A previous study found that approximately 40% of children who initiated ART before two and three months of age had negative HIV serology using a third‐generation HIV serology assay [10]. Factors predictive of seronegativity were higher CD4 percentage prior to ART initiation, no intermittent viraemia, and ART initiation prior to three months of age [10]. A randomized controlled trial using a fourth‐generation assay found 46% of HIV‐positive children who started ART before 12 weeks of age were seronegative by two years of age, compared to only 11% in children who started ART later. The authors concluded that HIV serology is not related to the status of HIV infection in children who received early ART [11]. Our results are consistent with these previous reports, with half of the infants in our cohort who initiated ART before three months being seronegative and a quarter who initiated ART between three and six months of age being seronegative.

Early initiation of ART can lead to a rapid decline in viral replication and improvement in host immune responses, leading to attenuated or defective virus [8, 13]. It is also possible that seronegativity may be linked to the lack of immune response in infants where viral suppression occurred before the developing immune system could recognize circulating viruses [14]. Early viral suppression has been associated with incomplete development of a HIV‐specific immune response [14, 15]. The majority of the children in our cohort were born before the era of universal ART, hence, were treated with ART later in the course of their disease. This may explain the low number of children who turned seronegative in our cohort (7.3%). The prevalence of seronegativity would expect to rise as more children are treated earlier.

Though not statistically significant, seronegative children did have higher nadir CD4 cell counts and CD4 cell counts at time of diagnosis, reflecting the earlier state of HIV infection at the time of ART initiation. This finding is in agreement with other studies that found a higher CD4 percentage prior to ART initiation was predictive of seronegativity [10].

We used a fourth‐generation HIV serology test in our study. While the fourth‐generation tests have been proven to better at detecting very early HIV infection, a study has found decreased sensitivity of fourth‐generation tests in detecting fading antibody in adults who received effective ART during acute infection [16]. However, a separate study at our centre found that the fourth‐generation assay can detect low levels of antibody which may result in a longer time to detect seroreversion in HIV‐exposed uninfected infants [12]. Thus, it is unlikely that using the fourth‐generation assay led to an overestimation of the seronegative rate in our study.

Seronegativity may reflect the smaller size of CD4 cells infected with HIV and the overall viral reservoir [17]. These individuals could potentially become post‐treatment controllers and potentially allow a functional cure [18, 19, 20, 21]. However, more studies are needed to ascertain such a hypothesis and to develop safe treatment interruption strategies. The PHIV individuals receiving ART reaching adulthood should be counselled on the potentially negative results if they seek out HIV testing with other clinicians who do not know their treatment history. Unplanned treatment interruptions will likely result in uncontrolled viral replication, immune deficiency, and increased risked of opportunistic infections.

This study is the first study reporting the frequency of seronegativity and the factors that potentially contribute to seronegativity in children, adolescents and young adults with PHIV in Asia. A seronegative test result can potentially eliminate stigmatization in some certain situations; however, seronegative may also lead to inappropriate treatment interruptions.

There are several limitations to our study. This was a small single‐centred study and fourth‐generation assays were used to test HIV serology, therefore, the results may not reflect paediatric populations in other settings. Due to infrequent VL monitoring in routine practice in Thailand, the time to viral suppression was longer than that reported in other studies. Clearly, the exact duration from ART initiation to viral suppression was certainly shorter than the time to first test report of viral suppression; however, both seronegative and seropositive groups had similar frequencies of routine VL monitoring.

## 4 CONCLUSIONS

Seroreversion or seronegativity is not rare in children and adolescents with PHIV. Early initiation of ART in infancy and shorter time to viral suppression after initiation of ART may predict seronegativity in children and adolescents with PHIV who are receiving effective ART. Seronegativity or seroreversion does not equal to HIV cure. Withholding or discontinuing ART could lead to viral rebound and disease progression. On the other hand, seronegativity may help reduce stigmatization, particularly when HIV testing is performed for other non‐medical purposes. Further study is required to prove the association between seronegativity and post‐treatment virological control.

## COMPETING INTERESTS

All authors declare no conflict of interest.

## AUTHORS’ CONTRIBUTIONS

KL, KC and PW designed the research study. PW, KL, NK and BK established the cohort including subject finding, invitation and inform consent process. PW, KL, SR, OW and WP performed the study. PW, KL and AM analysed the data. PW, KL and KC wrote the primary draft of the manuscript. All authors contributed to subsequent drafts and approved the final manuscript.
